# Supplementary material for: Stress granules counteract senescence by sequestration of PAI‐1
Source: EMBO Rep. 2018 Mar 29;19(5):e44722. doi: 10.15252/embr.201744722 (PMC5934773; doi:10.15252/embr.201744722)
Supplement: Supplementary file 1 — Appendix [file EMBR-19-e44722-s001.pdf]

Appendix

Table of Contents

Appendix Figure S1 - Expression of T-antigen decreases during the senescence of IDH4 cells and no chromosomal abnormalities detected in WI-38 fibroblasts. p2

Appendix Figure S2 - Repeated exposure to arsenite decreased the number of senescent WI-38 fibroblasts without affecting the levels of key protein factors involved in senescence. p3

Appendix Figure S3 - The assembly of stress granules in senescing WI-38 fibroblasts decreases upon repeated exposure to arsenite. p4

Appendix Figure S4 - Stress granules completely disassemble in IDH4 and WI-38 cells 24 hrs post treatment with arsenite. p5

Appendix Figure S5 - Repeated exposure to arsenite for three days is sufficient to decrease the number of senescent IDH4 and WI-38 cells. p6

Appendix Figure S6 - Cycloheximide prevents the assembly of stress granules independently of its effect on general translation. p7

Appendix Figure S7 - Puromycin (Puro) does not impair stress granule formation or the arsenite mediated decrease in number of senescent cells. p8

Appendix Figure S8 - Impairment of Autophagy or the Stress Response does not reverse the arsenite mediated decrease in the number of senescent cells. p9

Appendix Figure S9 - Impairment of Proteasome function does not reverse the arsenite mediated decrease in the number of senescent cells. p10

Appendix Figure S10 - PAI-1 co-localizes to stress granules after arsenite treatment in WI-38 cells. p11

Appendix Figure S11 - Stress granule formation inhibits secretion of PAI-1. P12

Appendix Figure S12 - Supplementation of media with recombinant PAI-1 reverses the effect of arsenite and Pateamine A on the senescence of WI-38 cells. p13

Appendix Figure S13 - Arsenite-induced formation of stress granules results in the activation of the cyclin D1/RB-pathway in WI-38 cells. p14

Appendix Figure Legends p15-19

Appendix Figure S1

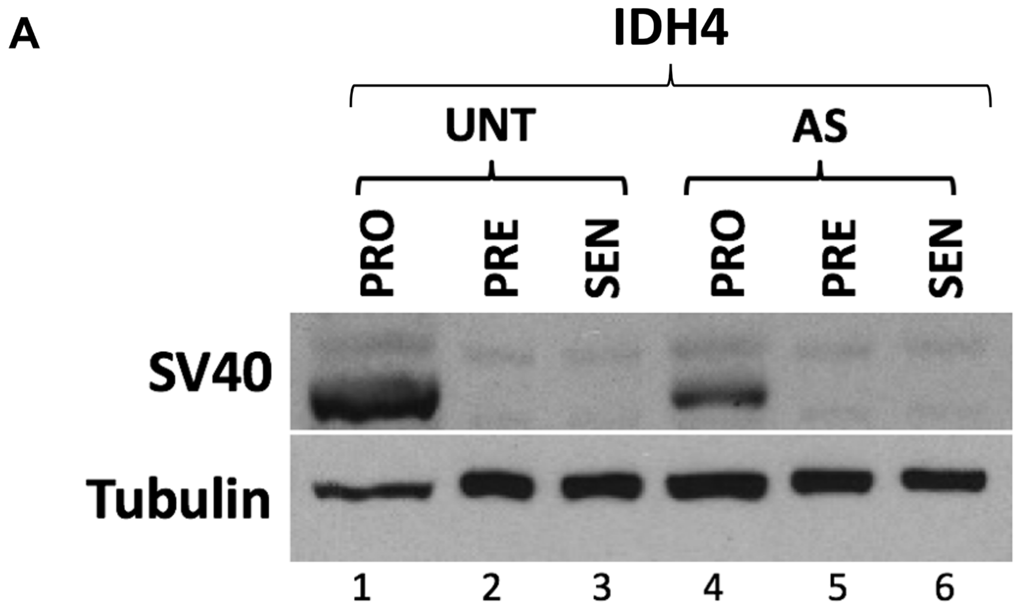

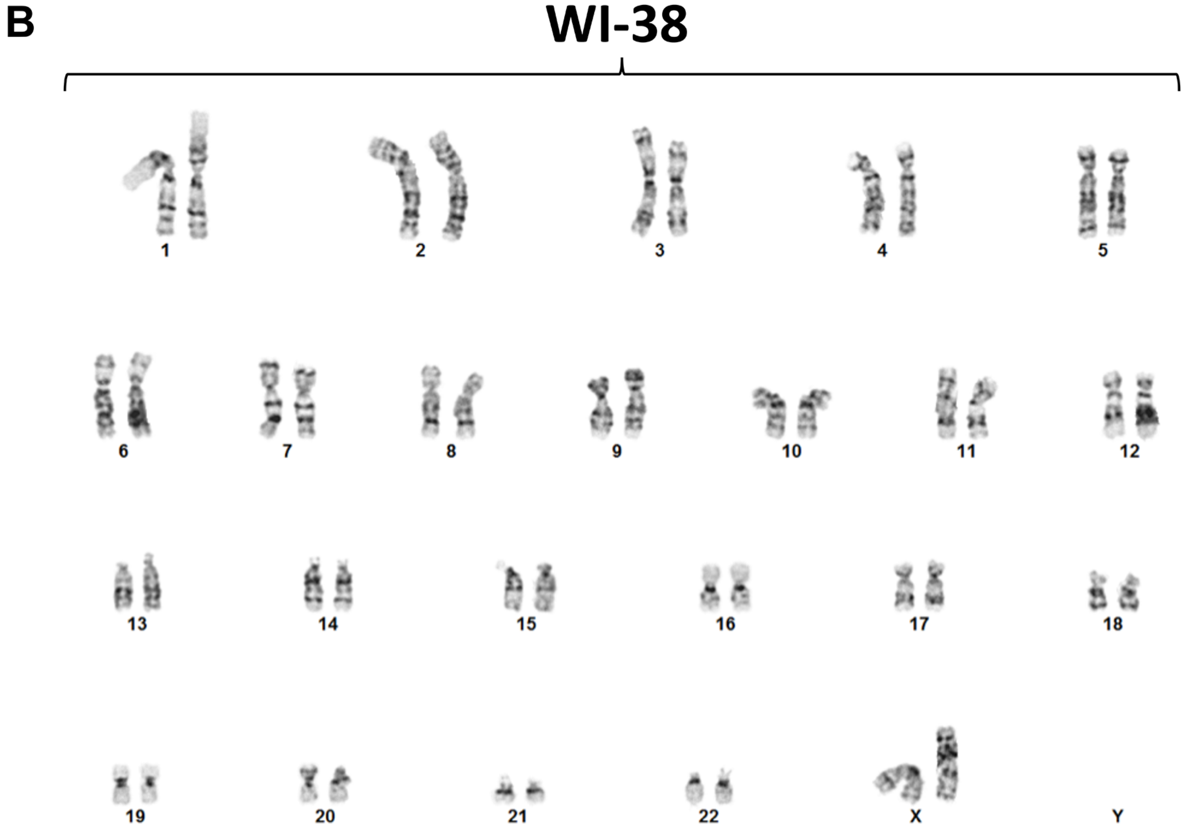

Appendix Figure S2

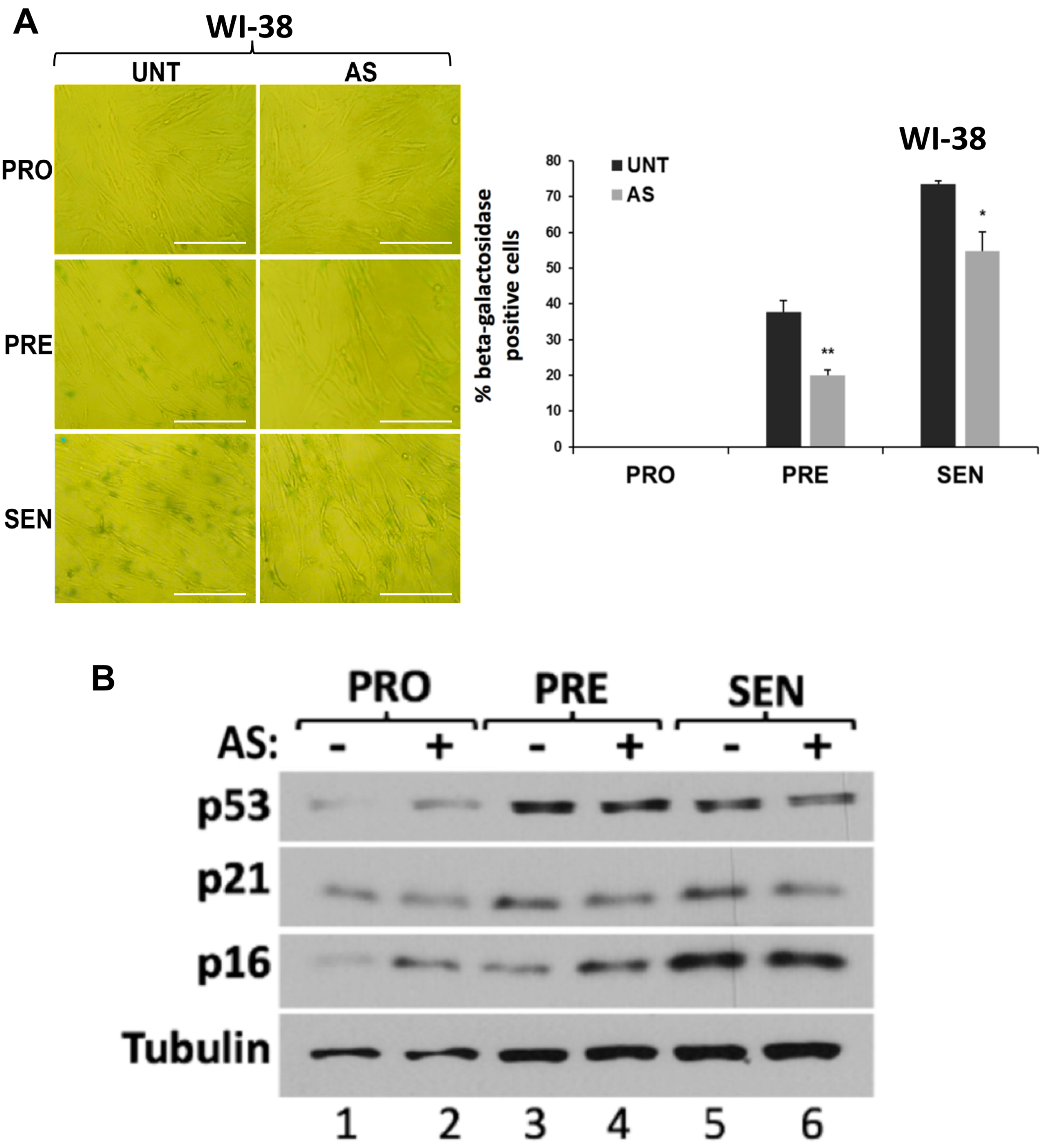

Appendix Figure S3

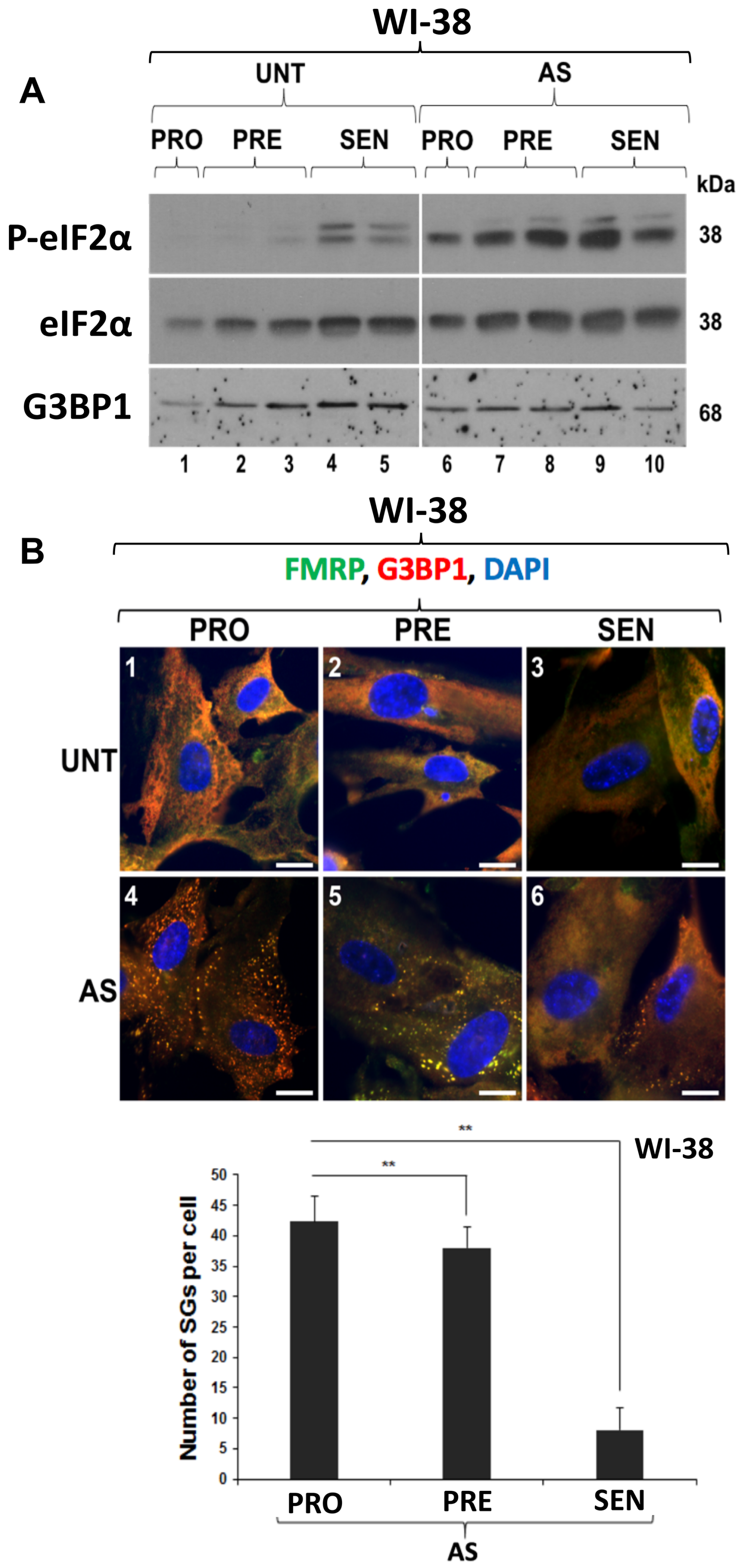

Appendix Figure S4

A

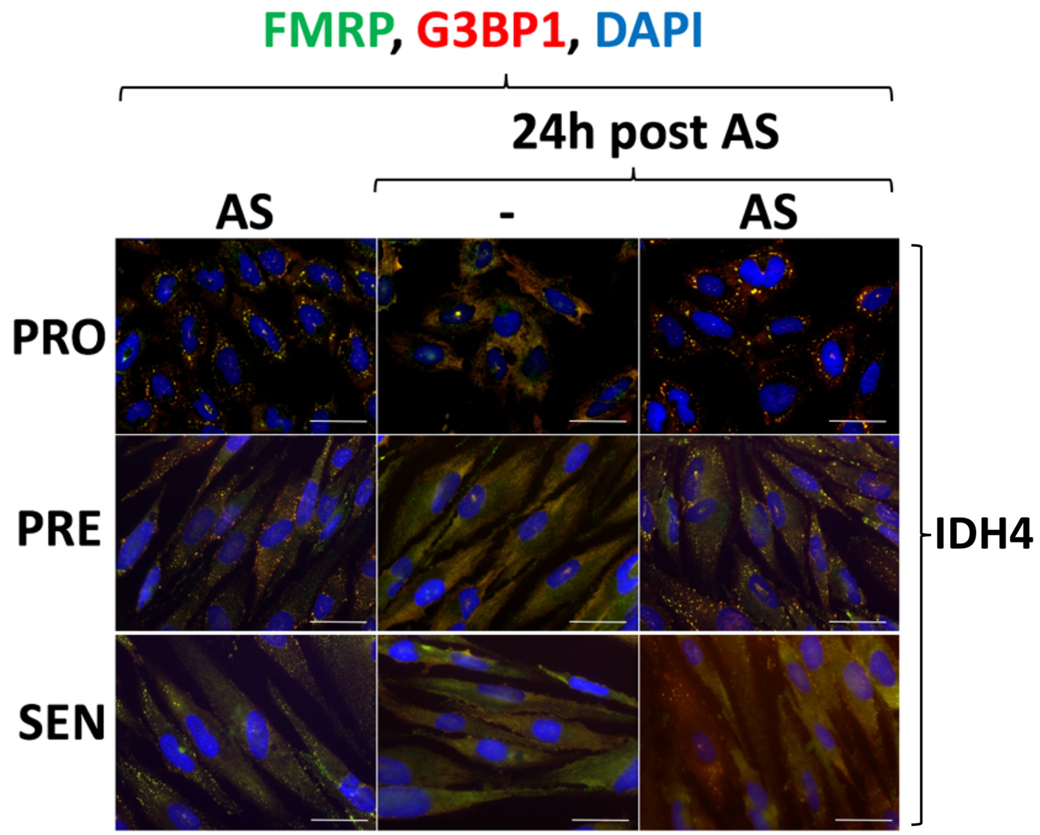

B

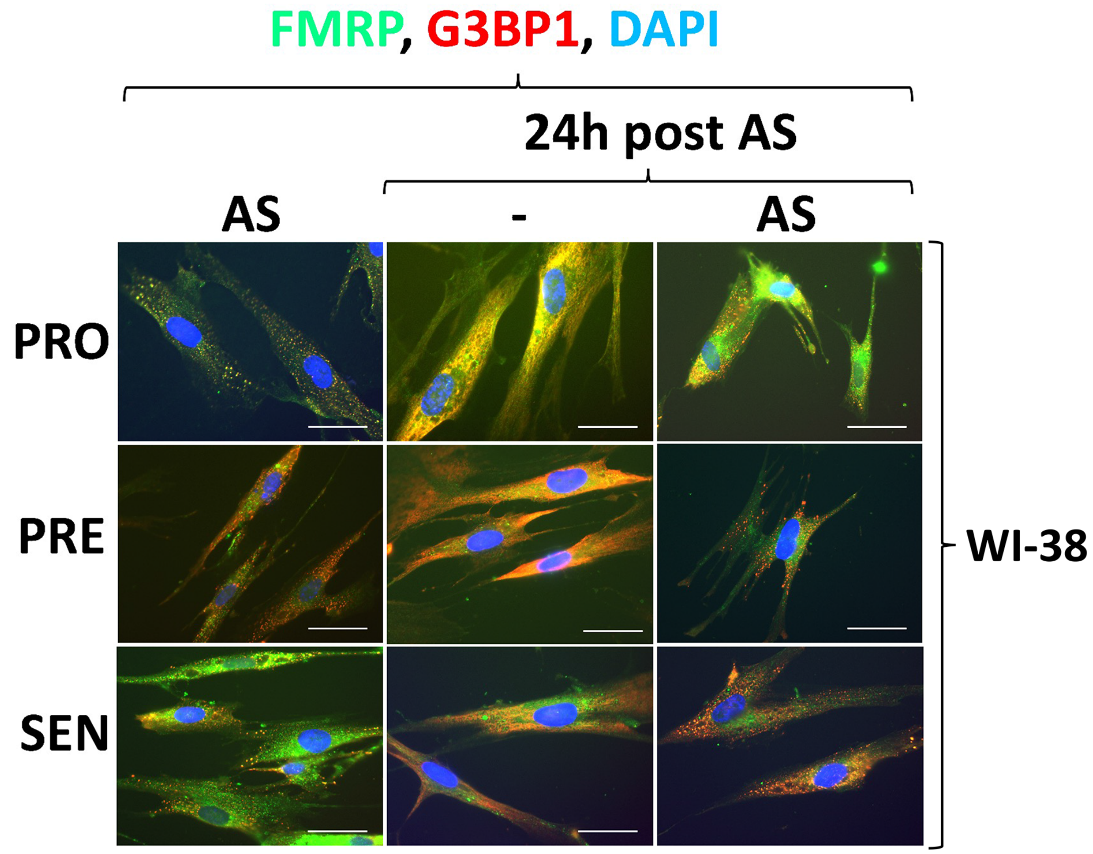

Appendix Figure S5

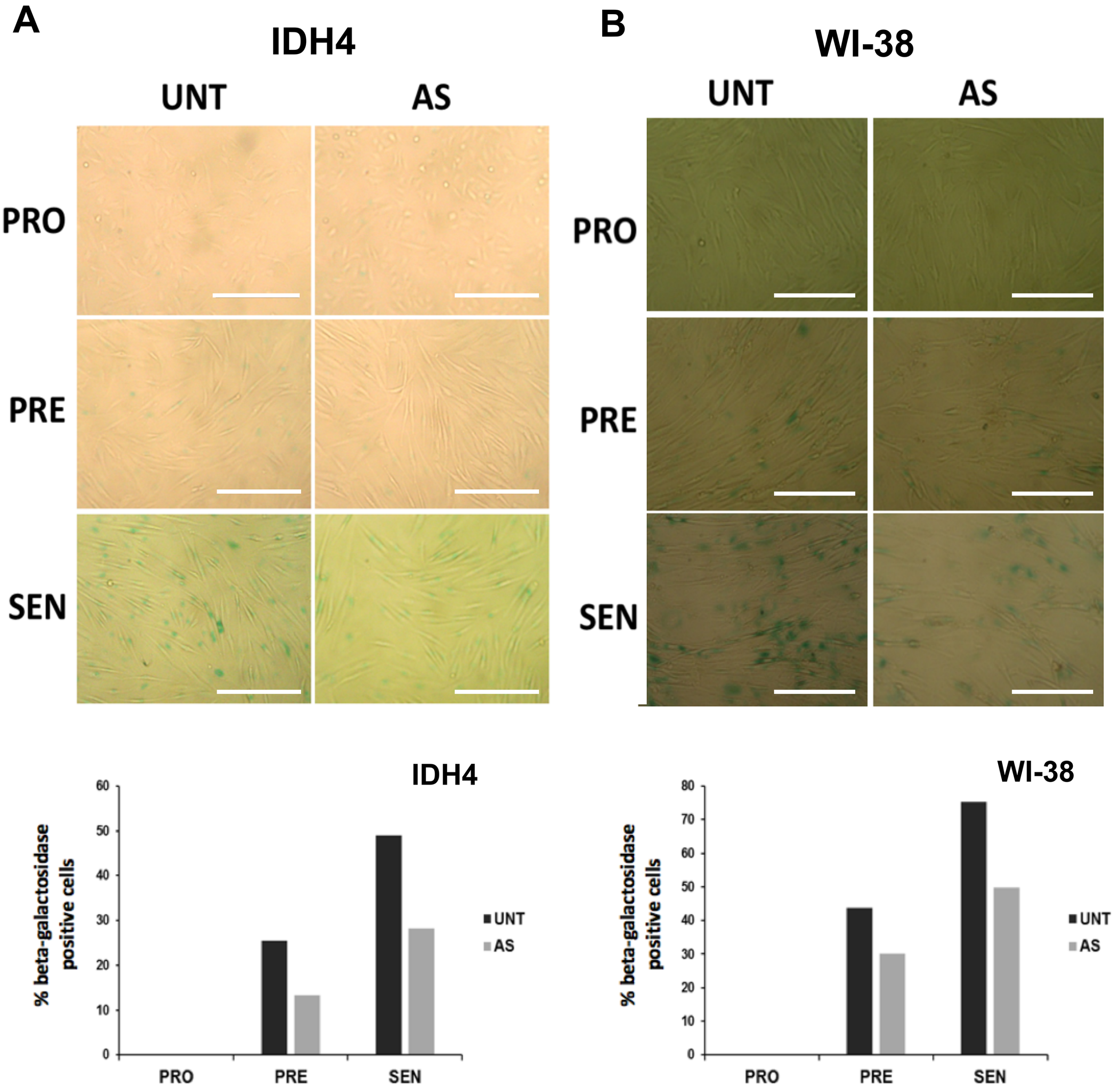

Appendix Figure S6

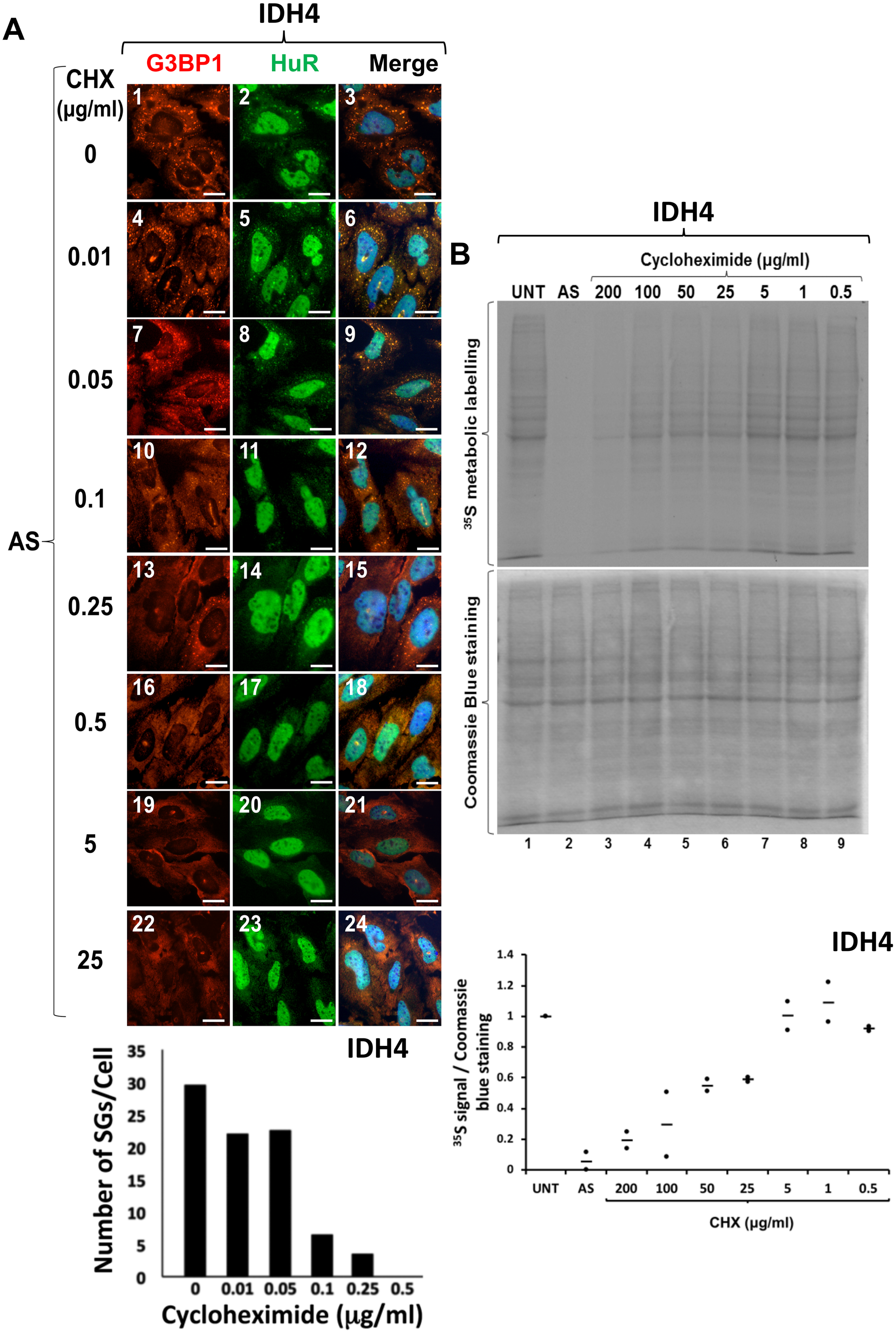

Appendix Figure S7

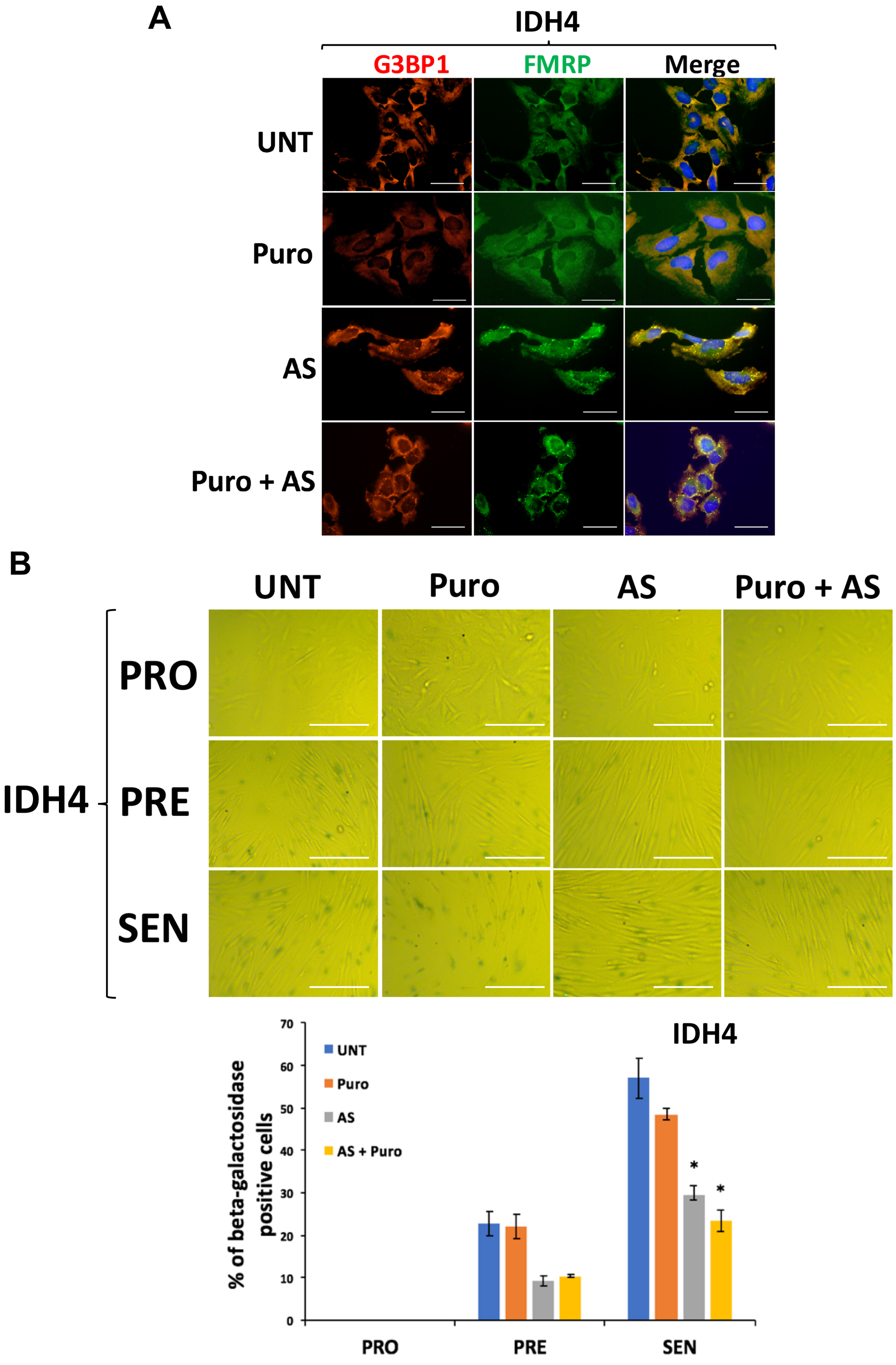

Appendix Figure S8

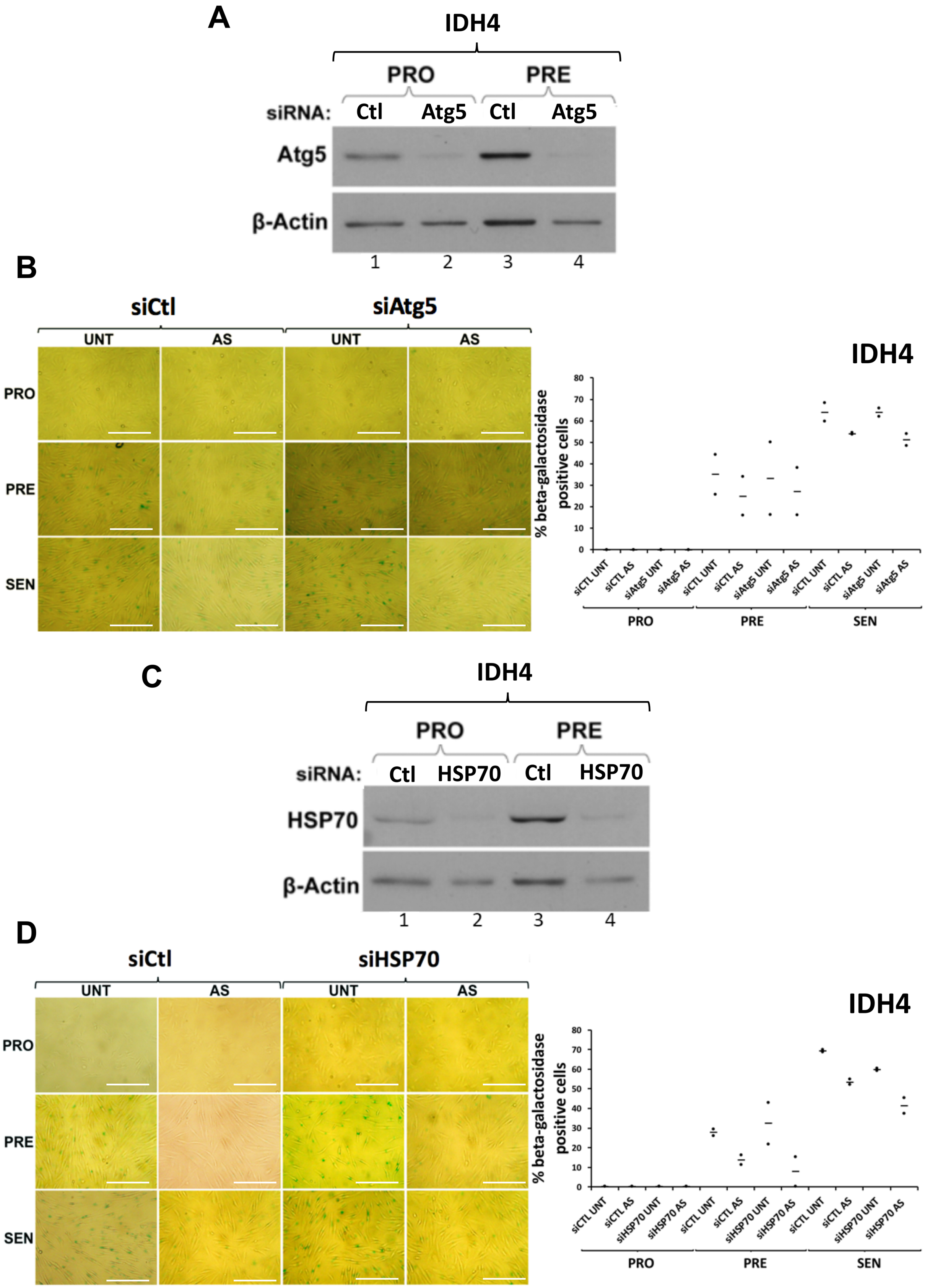

Appendix Figure S9

A

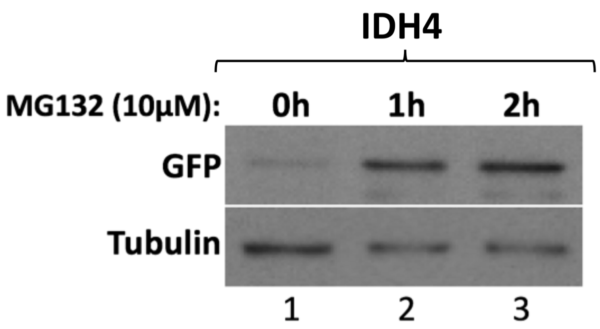

B

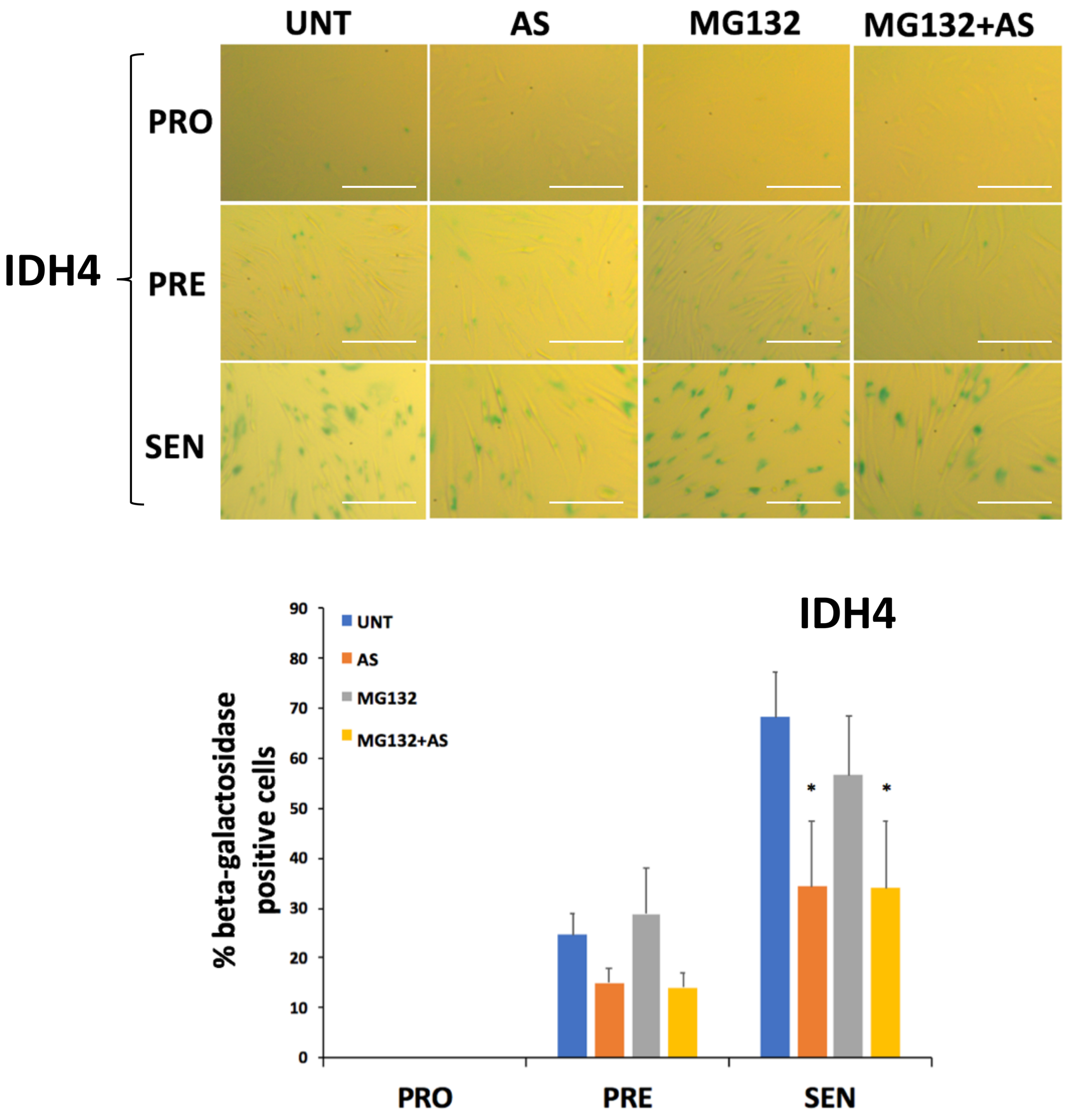

Appendix Figure S10

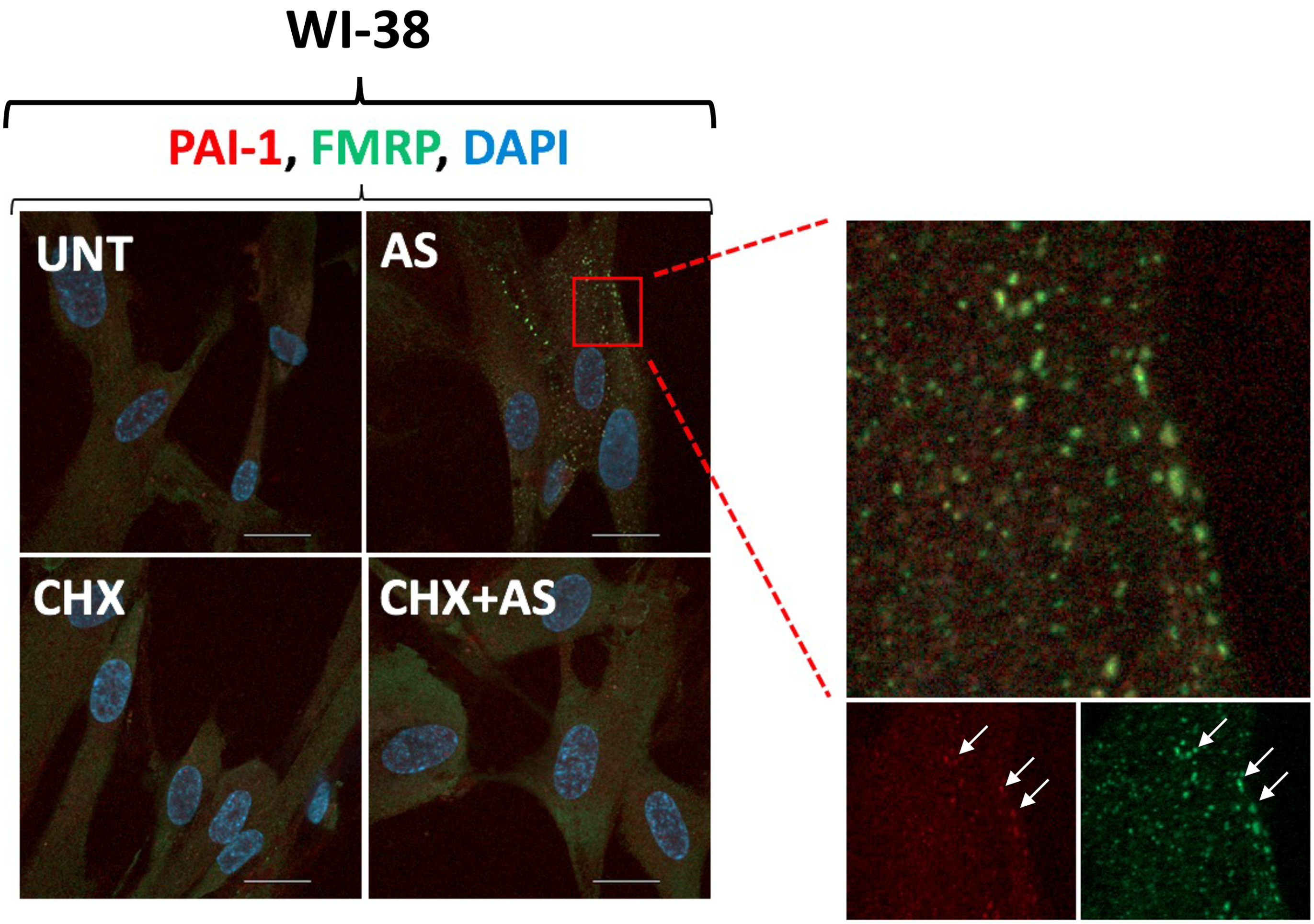

Appendix Figure S11

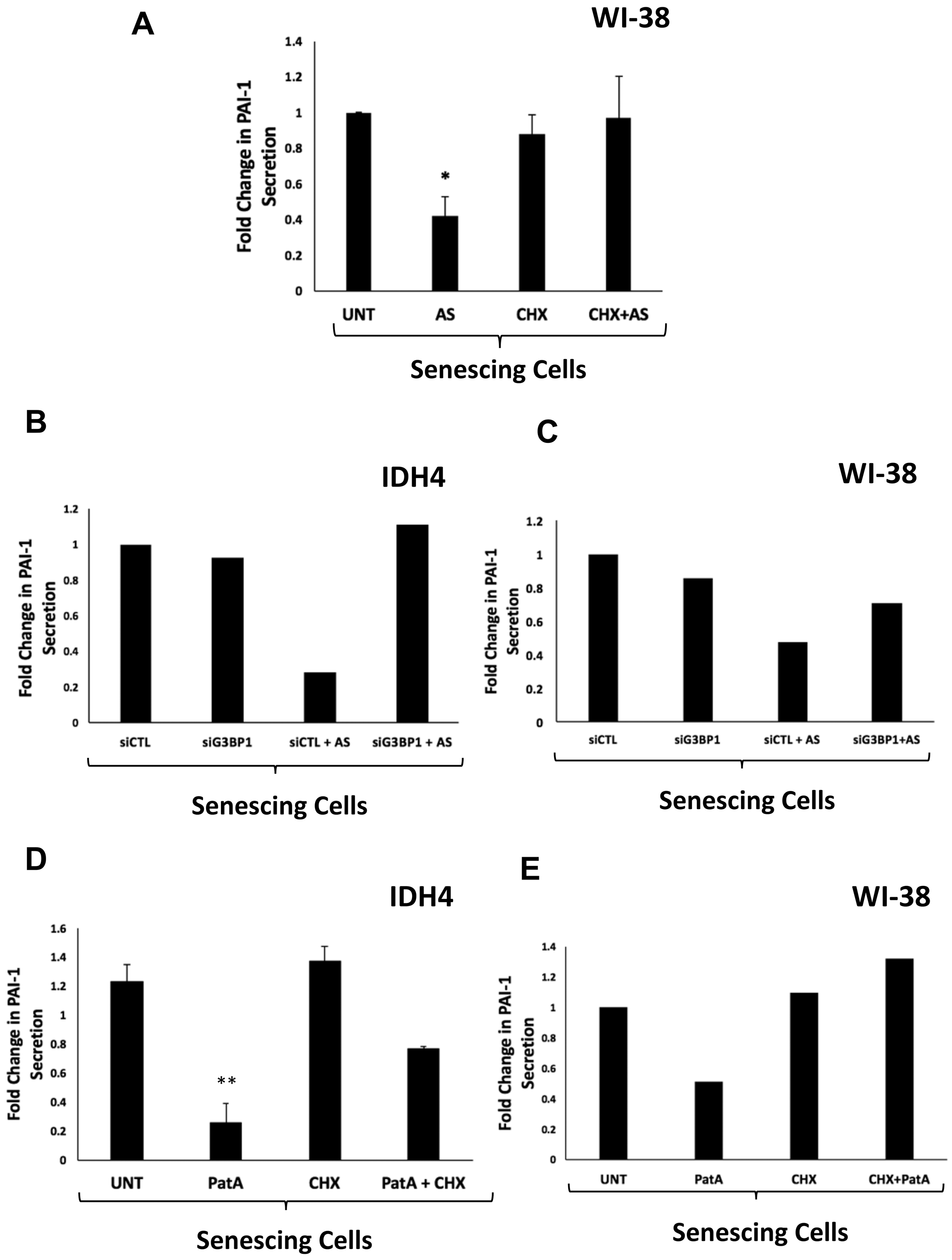

Appendix Figure S12

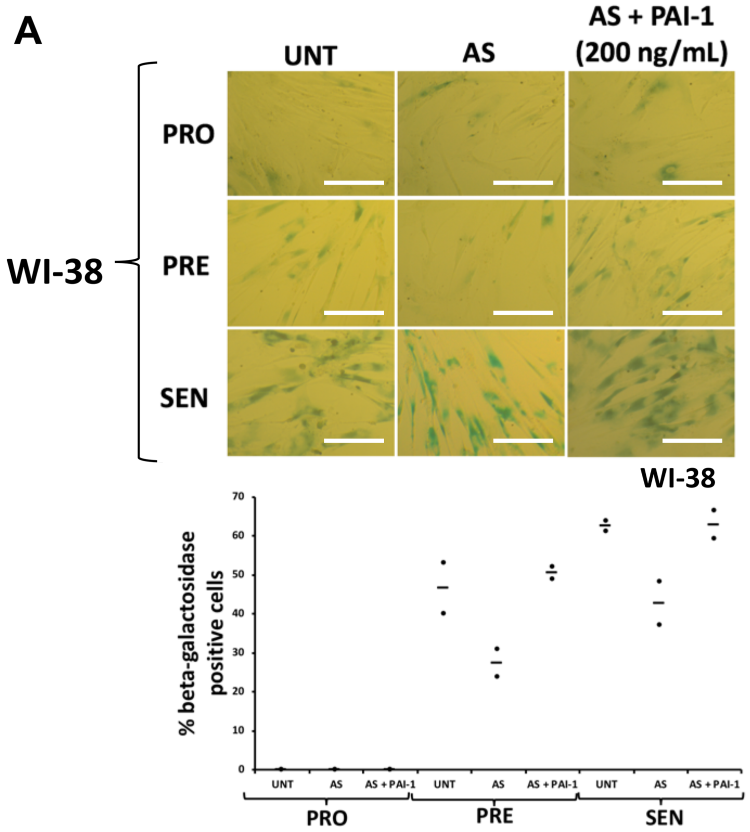

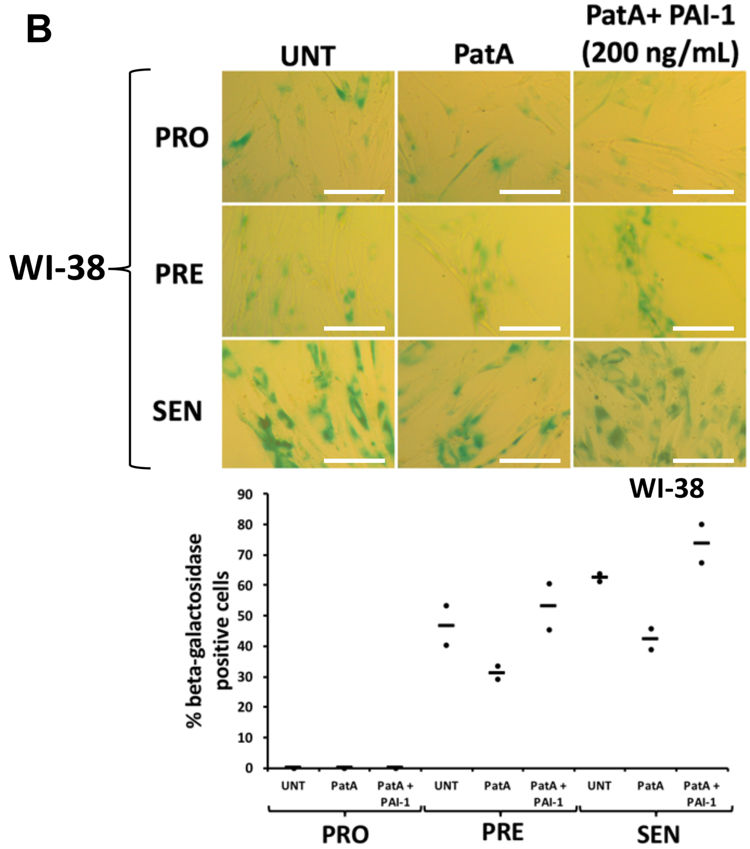

Appendix Figure S13

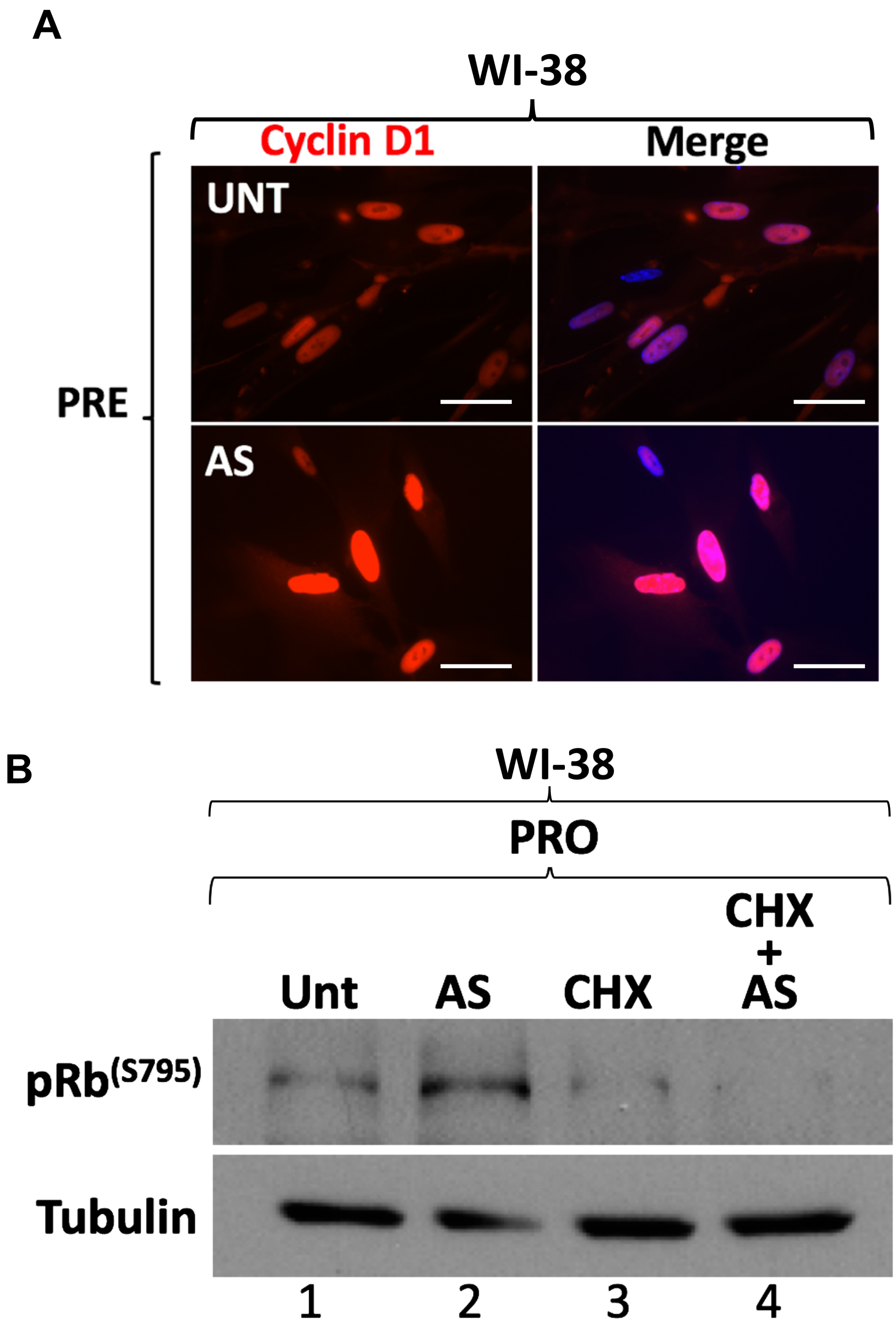

Appendix Figure Legends

Appendix Figure S1 - Expression of T-antigen decreases during the senescence of IDH4 cells and no chromosomal abnormalities detected in WI-38 fibroblasts.

A Whole-cell extracts from IDH4 cells treated daily post-induction of senescence for 30 min with (AS) or without (UNT) 0.5mM sodium arsenite were prepared and analyzed by western blot using antibodies for SV40 T-antigen and tubulin (loading control).

B Representative metaphase (46,XX) of WI-38 human lung fibroblasts reveal no chromosomal abnormalities. Metaphase preparation and GTG banding techniques were performed according to standard cytogenetic procedures.

Appendix Figure S2 - Repeated exposure to arsenite decreased the number of senescent WI-38 fibroblasts without affecting the levels of key protein factors involved in senescence.

A (left) WI-38 cells were treated daily post-induction of senescence for 30min with (AS) or without (UNT) 0.5 mM sodium arsenite. Proliferating (PRO), presenescent (PRE) and senescent (SEN) cells were subsequently subjected to staining for β-galactosidase activity. Phase contrast images demonstrating the staining of the WI-38 cells at the various stages of the senescence process. Scale bars, 400 µm. (right) Graph represents the percentage of cells that stained positive for β-galactosidase activity (stained blue-green) in the phase contrast images shown in (left panel). The percentage of senescent cells in each experiment was calculated using three random fields.

B Whole-cell extracts from WI-38 cells at the PRO, PRE and SEN stages treated (AS) or not (UNT) with sodium arsenite as indicated above were prepared and analyzed by western blot using antibodies for p53, p21, p16 and tubulin (loading control).

Data Information: In (A) data are represented as a mean of three independent experiments ± SE (*error bars*). *$p<0.05$, **$p<0.01$ (Student's t-test).

Appendix Figure S3 - The assembly of stress granules in senescing WI-38 fibroblasts decreases upon repeated exposure to arsenite.

WI-38 cells were treated daily for 30min with or without 0.5mM sodium arsenite (AS) post-induction of senescence.

A Whole-cell extracts from WI-38 cells treated as indicated above were prepared and western blot analysis was performed using antibodies for P-eIF2α, eIF2α and G3BP1 (loading control).

B (top) After treatment, cells at different stages of senescence (PRO, PRE, SEN), were fixed, permeablized, and analyzed by immunofluorescence with antibodies against the SG markers FMRP and G3BP1. DAPI staining was used to visualize the nuclei of the cells. Bars, 20µm. (bottom) Graph represents the number of SGs in the immunofluorescence images presented in (top panel). The average number of SGs per cell was calculated by normalizing the total number of SGs in each field to the total number of cells. Three random fields were used for each quantification.

Data Information: In (B) data are represented as a mean of three independent experiments ± SE (*error bars*). **p<0.01 (Student's t-test).

Appendix Figure S4 - Stress granules completely disassemble in IDH4 and WI-38 cells 24 hrs post treatment with arsenite.

A IDH4 cells were treated with 0.5 mM sodium arsenite for 30min daily during senescence. Cells were fixed 30 min after treatment, 24 hrs after removing arsenite from the growth media, as well as 30 min after treatment of recovered cells. Immunofluorescence experiments were performed using antibodies against G3BP1 and FMRP. DAPI staining was used to visualize the nuclei of the cells. Bars, 50µm.

B WI-38 cells were treated with 0.5 mM sodium arsenite for 30min daily during senescence. Cells were fixed 30 min after treatment, 24 hrs after removing arsenite from the growth media, as well as 30 min after treatment of recovered cells. Immunofluorescence experiments were performed using antibodies against G3BP1 and FMRP. DAPI staining was used to visualize the nuclei of the cells. Bars, 50µm.

Appendix Figure S5 - Repeated exposure to arsenite for three days is sufficient to decrease the number of senescent IDH4 and WI-38 cells.

A (top) IDH4 cells were treated with or without 0.5mM sodium arsenite daily for three days post-induction of senescence. Cells at the PRO, PRE, and SEN stages of senescence were fixed and stained for β-galactosidase activity. Phase contrast images demonstrating the staining of the IDH4 cells at the various stages of the senescence process are shown. Scale bars, 400 µm. (bottom) Graph represents the percentage of cells that stained positive for β-galactosidase activity (stained blue-green) in the phase contrast images shown in (top panel). The percentage of senescent cells in each experiment was calculated using three random fields.

B WI-38 cells were treated as described in (A) for three days post-induction of senescence. Cells at the PRO, PRE, and SEN cells were subsequently subjected to staining for β-galactosidase activity (top) and analyzed as described above for IDH4 cells (bottom).

Data Information: In (A and B) data are representative of one experiment.

Appendix Figure S6 - Cycloheximide prevents the assembly of stress granules independently of its effect on general translation.

A (top) IDH4 cells were treated for 30 min with or without 0 – 25 µg/ml of cycloheximide (CHX). Immunofluorescence experiments were performed using antibodies against G3BP1 and HuR. DAPI staining was used to visualize the nuclei of the cells. Bars, 40 µm. (bottom) Graph represents the number of SGs in the immunofluorescence images presented in the top panels. The average number of SGs per cell was calculated by normalizing the total number of SGs in each field to the total number of cells.

B (top) IDH4 cells were treated for 30 min with or without 0.5 mM sodium arsenite or 0.5 – 200

μg/ml of cycloheximide (CHX). Cells were then incubated with ^{35}S-methionine for 30 min and whole-cell extracts were separated on a polyacrylamide gel. ImageJ was used to quantify the intensity of the bands for ^{35}S labelling, which were normalized to the intensity of the bands from Coomassie Blue staining (loading control). (bottom) The graph represents the intensity of the ^{35}S labelling normalized to the intensity of the Coomassie Blue staining. Data are represented as a mean (represented by –) of two independent experiments.

Data Information: In (A) data are representative of one experiment. In (B) data are represented as a mean (represented by –) of two independent experiments.

Appendix Figure S7 - Puromycin (Puro) does not impair stress granule formation or the arsenite mediated decrease in number of senescent cells.

A After treatment with or without arsenite and/or puromycin, IDH4 cells at different stages of senescence (PRO, PRE, and SEN) were fixed, permeablized, and analyzed by immunofluorescence with antibodies against the SG markers FMRP and G3BP1. DAPI staining was used to visualize the nuclei of the cells. Bars, 50μm.

B (top) IDH4 cells were treated daily as described in (A). Cells at the PRO, PRE and SEN stage were subsequently subjected to staining for β-galactosidase activity. Phase contrast images demonstrating β-galactosidase staining are shown. Scale bars, 200 μm. (bottom) Graph represents the percentage of cells that stained positive for β-galactosidase activity (stained blue-green) in (top panel). The percentage of senescent cells in each experiment was calculated using three random fields.

Data Information: In (B) data are represented as a mean of three independent experiments ± SE (*error bars*). * $p<0.05$ (Student's t-test).

Appendix Figure S8 - Impairment of Autophagy or the Stress Response does not reverse the arsenite mediated decrease in the number of senescent cells.

A Proliferating IDH4 cells were transfected with a control (Ctl) or a Atg5-specific siRNA, and senescence was induced 24 hrs post-transfection. Whole-cell extracts from IDH4 cells treated as above were prepared and analyzed by western blot using antibodies for Atg5, and beta-actin (loading control).

B (left) Cells treated as described in (A) were treated daily with or without AS (0.5 mM) for 30 min daily and subsequently subjected to staining for β-galactosidase activity at the PRO, PRE, and SEN stages. Phase contrast images demonstrating β-galactosidase staining are shown. Scale bars, 400 μm. (right) Graph represents the percentage of cells that stained positive for β-galactosidase activity (stained blue-green) in (left panel). The percentage of senescent cells in each experiment was calculated using three random fields.

C Proliferating IDH4 cells were transfected with a control (Ctl) or a HSP70-specific siRNA, and senescence was induced 24 hrs post-transfection. Whole-cell extracts from these IDH4 cells, treated as described above, were prepared and analyzed by western blot using antibodies for HSP70, and beta-actin (loading control).

D (left) Cells treated as described in (C) were treated daily with or without AS (0.5 mM) for

30min daily and subsequently subjected to staining for β-galactosidase activity at the PRO, PRE, and SEN stages. Phase contrast images demonstrating β-galactosidase staining are shown. Scale bars, 400 μm. (right) Graph represents the percentage of cells that stained positive for β-galactosidase activity (stained blue-green) in (left panel). The percentage of senescent cells in each experiment was calculated using three random fields.

Data Information: In (B and D) data are represented as a mean (represented by –) of two independent experiments.

Appendix Figure S9 - Impairment of Proteasome function does not reverse the arsenite-mediated decrease in the number of senescent cells.

A GFPu cells (293 cells stably transfected with a GFP reporter construct fused to a proteasome targeting sequence were treated with 10μM MG132 for different periods of time. The expression of GFP protein was monitored by Western blot using the anti-GFP antibody to assess the inhibition of the proteasome activity.

B (top) IDH4 cells at the PRO, PRE and SEN stage were treated daily with or without AS (0.5 mM) for 30 min and/or pre-treated MG132 (10 μM) for 2 hours and subsequently subjected to staining for β-galactosidase activity and PRO, PRE, and SEN stages. Phase contrast images demonstrating β-galactosidase staining are shown. Scale bars, 200 μm. (bottom) Graph represents the percentage of cells that stained positive for β-galactosidase activity (stained blue-green) in (top panel). The percentage of senescent cells in each experiment was calculated using three random fields. Data are represented as a mean of three independent experiments ± SE (*error bars*). * $p<0.05$ (Student's t-test).

Appendix Figure S10 - PAI-1 co-localizes to stress granules after arsenite treatment in WI-38 cells.

WI-38 cells at the PRO stage were treated with or without sodium arsenite (AS) and/or cycloheximide (CHX) for 30 minutes, fixed, permeabilized and analyzed by immunofluorescence with an antibody specific for FMRP or PAI-1 proteins. DAPI staining was used to visualize the nuclei of the cells. The red square in the AS panel represents the area that was expanded and is shown on the right. The two panels below the expanded box show individual staining for PAI-1 (red) and FMRP (green). Arrows indicate examples of co-localized PAI-1 and FMRP in the same foci. Scale bars, 50μm.

Appendix Figure S11 - Stress granule formation inhibits secretion of PAI-1.

A Secreted PAI-1 levels in supernatant obtained from senescing WI-38 cells treated with or without arsenite (AS) in the presence or absence of cycloheximide (CHX) were assessed by ELISA.

B – C Proliferating IDH4 (B) and WI-38 (C) cells were transfected with a control (Ctl) or a G3BP1-specific siRNA, and senescence was induced 24 hrs post-transfection. Secreted PAI-1 levels in supernatant obtained from these cells treated with or without AS were assessed by ELISA.

D – E Secreted PAI-1 levels in supernatant obtained from senescing IDH4 (D) and WI-38 (E)

cells treated with PatA with or without CHX were assessed by ELISA.

Data Information: In (A) and (D) data are represented as a mean of three independent experiments ± SE (*error bars*). *$p<0.05$ (Student's t-test). In (B), (C) and (E) data are representative of one experiment.

Appendix Figure S12 - Supplementation of media with recombinant PAI-1 reverses the effect of arsenite and Pateamine A on the senescence of WI-38 cells.

A (top) WI-38 cells were treated daily during senescence for 30min with or without arsenite (AS). These cells were then supplemented daily or not with 200 ng/ml recombinant PAI-1. Cells at the PRO, PRE and SEN stage were subsequently subjected to staining for β-galactosidase activity. Phase contrast images demonstrating β-galactosidase staining are shown. Scale bars, 200 µm. (bottom) Graph represents the percentage of cells that stained positive for β-galactosidase activity (stained blue-green) in A and B was calculated using three random fields.

B (top) WI-38 cells were treated with a single dose during senescence for 30min with or without Pateamine A (PatA). Cells were then supplemented daily or not with 200 ng/ml recombinant PAI-1. Cells at the PRO, PRE and SEN stage were subsequently subjected to staining for β-galactosidase activity. Phase contrast images demonstrating β-galactosidase staining are shown. Scale bars, 200 µm. (bottom) Graph represents the percentage of cells that stained positive for β-galactosidase activity (stained blue-green) in A and B was calculated using three random fields.

Data Information: In (A) and (B) data are represented as a mean of a minimum of two independent experiments ± SE (*error bars*). *$p<0.05$ (Student's t-test).

Appendix Figure S13 - Arsenite-induced formation of stress granules results in the activation of the cyclin D1/RB-pathway in WI-38 cells.

A Cells at the PRO and PRE stages were fixed, permeabilized and analyzed by immunofluorescence with an antibody specific for Cyclin D1. DAPI staining was used to visualize the nuclei of the cells. Bars, 50 µm.

B Western blots were performed using whole-cell extracts from PRO cells, and antibodies specific for pRb (S795) and Tubulin (control) proteins.
