# Supplementary material for: Stress granules counteract senescence by sequestration of PAI‐1
Source: EMBO Rep. 2018 Mar 29;19(5):e44722. doi: 10.15252/embr.201744722 (PMC5934773; doi:10.15252/embr.201744722)
Supplement: Supplementary file 2 — Expanded View Figures PDF [file EMBR-19-e44722-s002.pdf]

Expanded View Figures

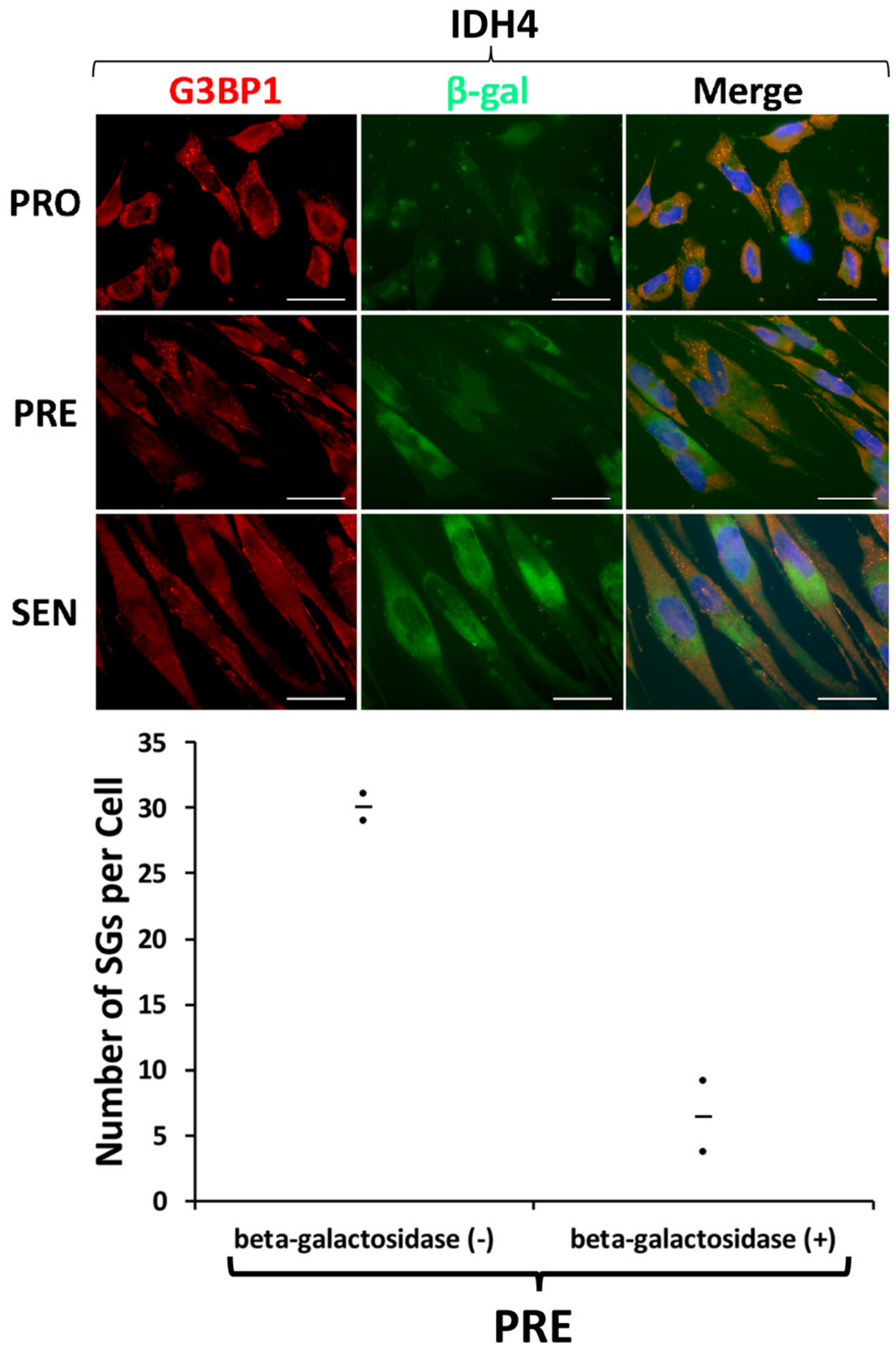

Figure EV1. β-galactosidase-positive cells contain fewer stress granules.
(top) IDH4 cells were fixed during PRO, PRE, and SEN phases of senescence, permeabilized, and analyzed by immunofluorescence using antibodies against G3BP1 and staining for hydrolyzed C_{12}FDG molecules (representing β-galactosidase activity). Scale bars, 50 μm. (bottom) Stress granules from the PRE phase were quantified and divided into β-galactosidase-positive (−) and β-galactosidase (+) groups. Data are represented as a mean (represented by −) of two independent experiments.

© 2018 The Authors

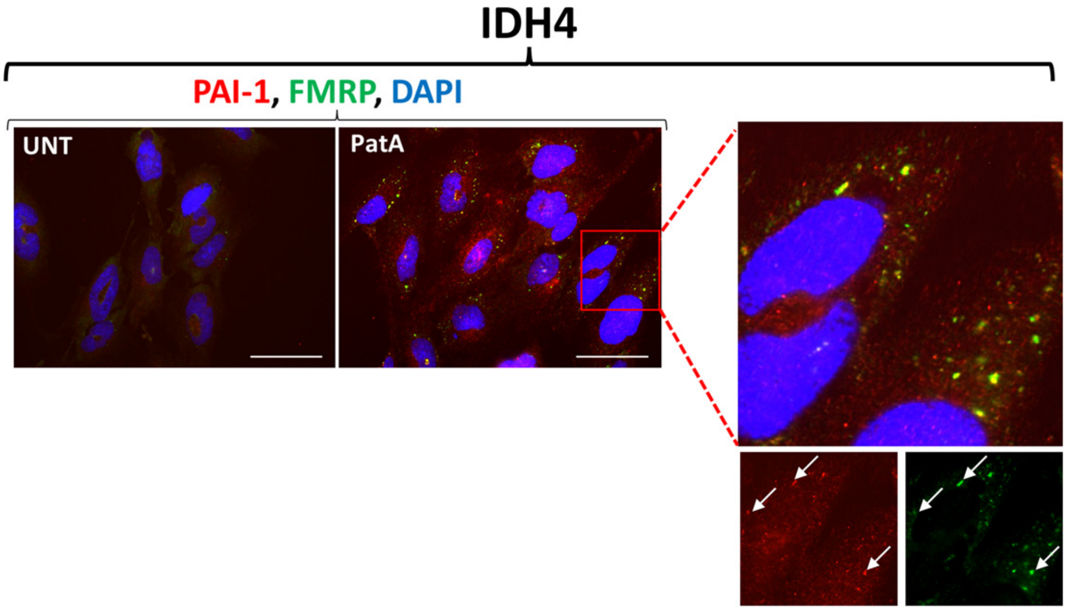

Figure EV2. PAI-1 co-localizes to stress granules after pateamine A treatment in IDH4 cells.
IDH4 cells were treated with 0.0125 μM pateamine A for 30 min, fixed, permeabilized, and analyzed by immunofluorescence with an antibody specific for FMRP or PAI-1 proteins. The red square in the AS panel represents the area that was expanded and is shown on the right. The two panels below the expanded box show individual staining for PAI-1 (red) and FMRP (green). Arrows indicate examples of co-localized PAI-1 and FMRP in the same foci. Scale bars, 50 μm.

 © 2018 The Authors

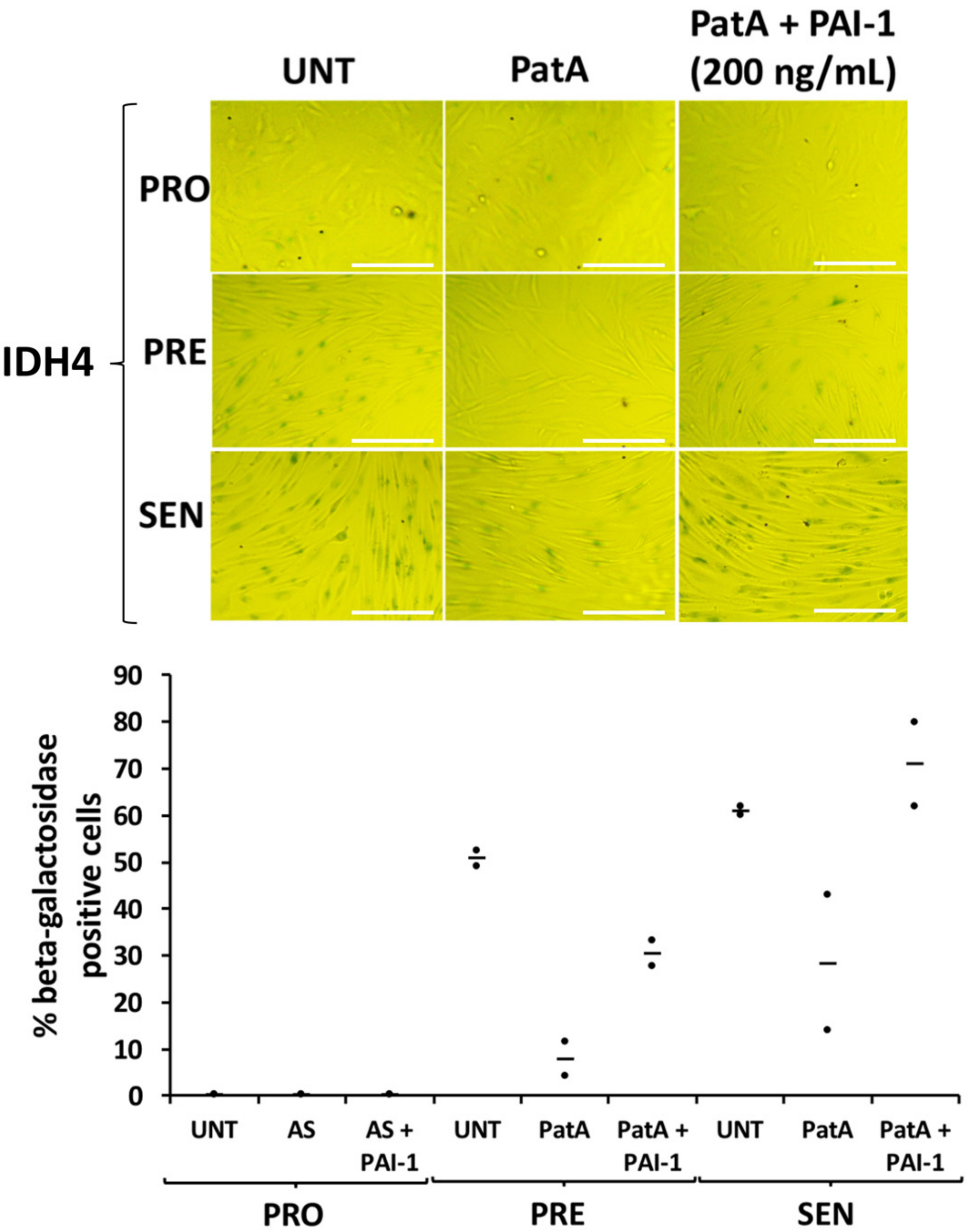

Figure EV3. Supplementation of media with recombinant PAI-1 reverses the effect of pateamine A on the senescence process.
(top) IDH4 (PRO) cells were treated for 30 min with a single dose of 0.0125 μM pateamine A (PatA), with and without supplementation of PAI-1 (200 ng/ml). Cells at the PRO, PRE, and SEN stages were subsequently subjected to staining for β-galactosidase activity. Phase contrast images demonstrating β-galactosidase staining are shown. (bottom) Graph represents the percentage of cells that stained positive for β-galactosidase activity (stained blue-green) in (top panel). Scale bars, 200 μm. The percentage of senescent cells in each experiment was calculated using three random fields. Data are represented as a mean (represented by –) of two independent experiments.

© 2018 The Authors

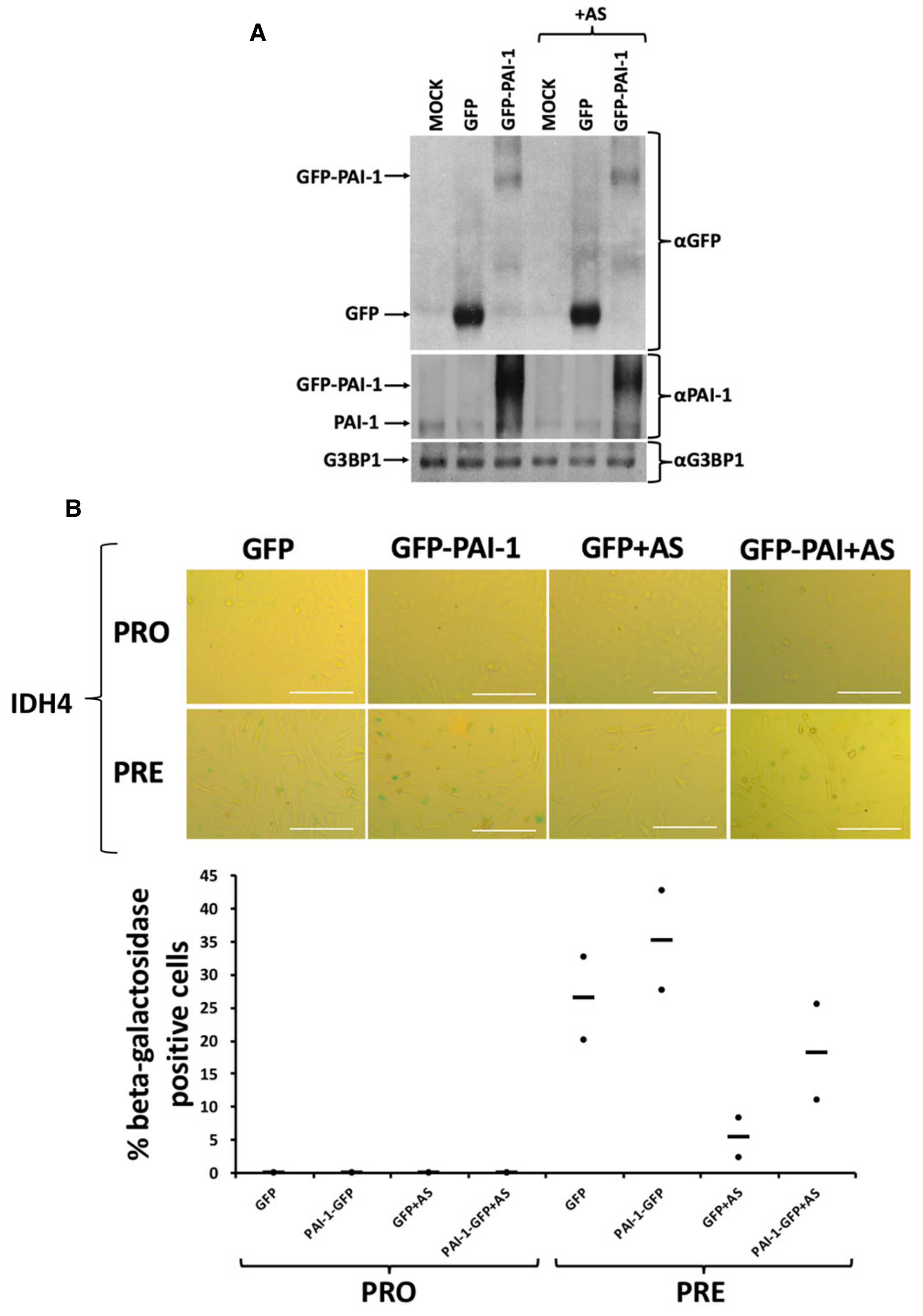

Figure EV4. Overexpression of PAI-1 reverses the arsenite-mediated effect on senescence.

IDH4 cells were transfected with a GFP control plasmid or a plasmid expressing GFP-fused to full-length human PAI-1. The transfected cells were treated daily for 30 minutes with or without AS post-induction of senescence.

A Whole-cell extracts from the IDH4 cells treated as described above and harvested at the PRO stage were prepared and analyzed by Western blot using antibodies for PAI-1, GFP, and G3BP1 (loading control).

B (top) Cells at the PRO and PRE stages were subsequently subjected to staining for β-galactosidase activity. Phase contrast images demonstrating β-galactosidase staining are shown. Scale bars, 200 μm. (bottom) Graph represents the percentage of cells that stained positive for β-galactosidase activity (stained blue-green) in (top panel). Data are represented as a mean (represented by –) of two independent experiments.

 © 2018 The Authors
