# Supplementary figures and images for: Stress granules counteract senescence by sequestration of PAI‐1
Source: EMBO Rep. 2018 Mar 29;19(5):e44722. doi: 10.15252/embr.201744722 (PMC5934773; doi:10.15252/embr.201744722)

Figure 1

Figure 1B

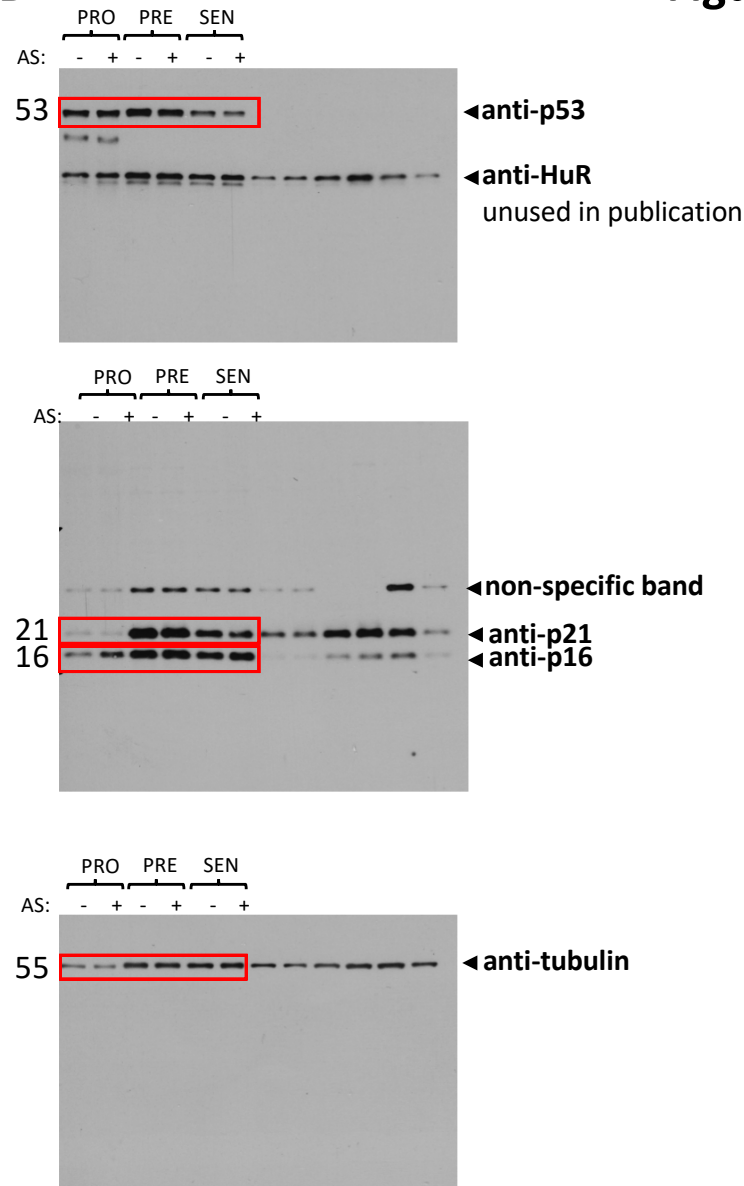

Figure 1C

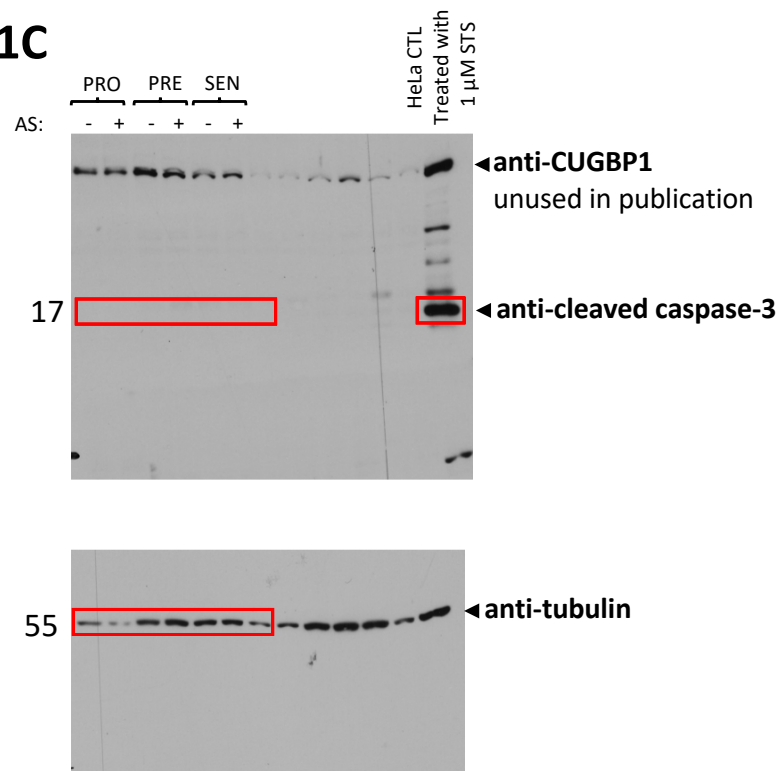

Supplement: Supplementary file 6 — Source Data for Figure 1 [file EMBR-19-e44722-s005.pdf]

Figure 2

Figure 2A

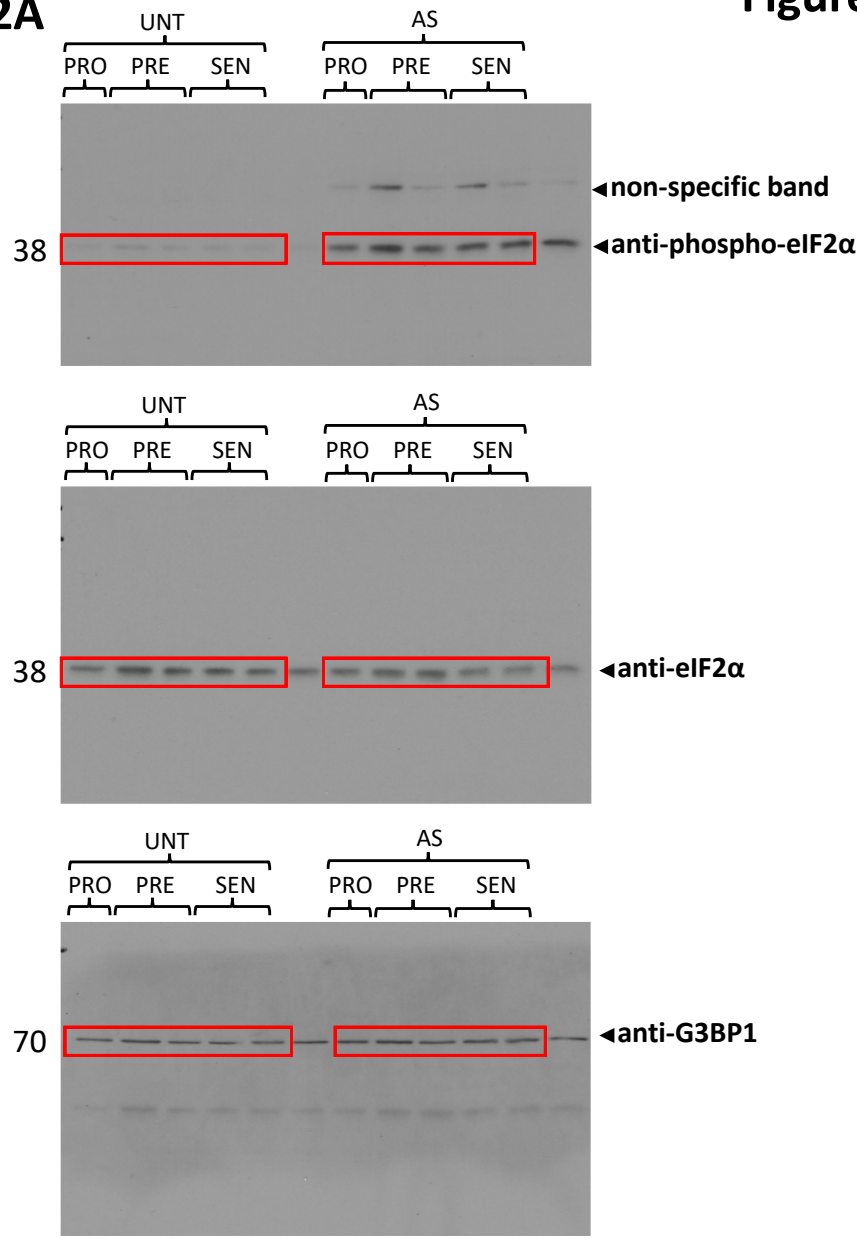

Figure 2B

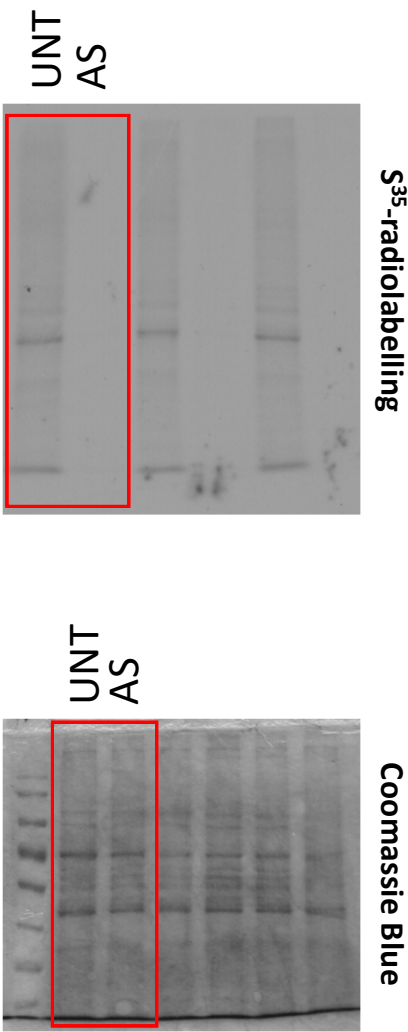

Supplement: Supplementary file 7 — Source Data for Figure 2 [file EMBR-19-e44722-s006.pdf]

Figure 3

Figure 3A

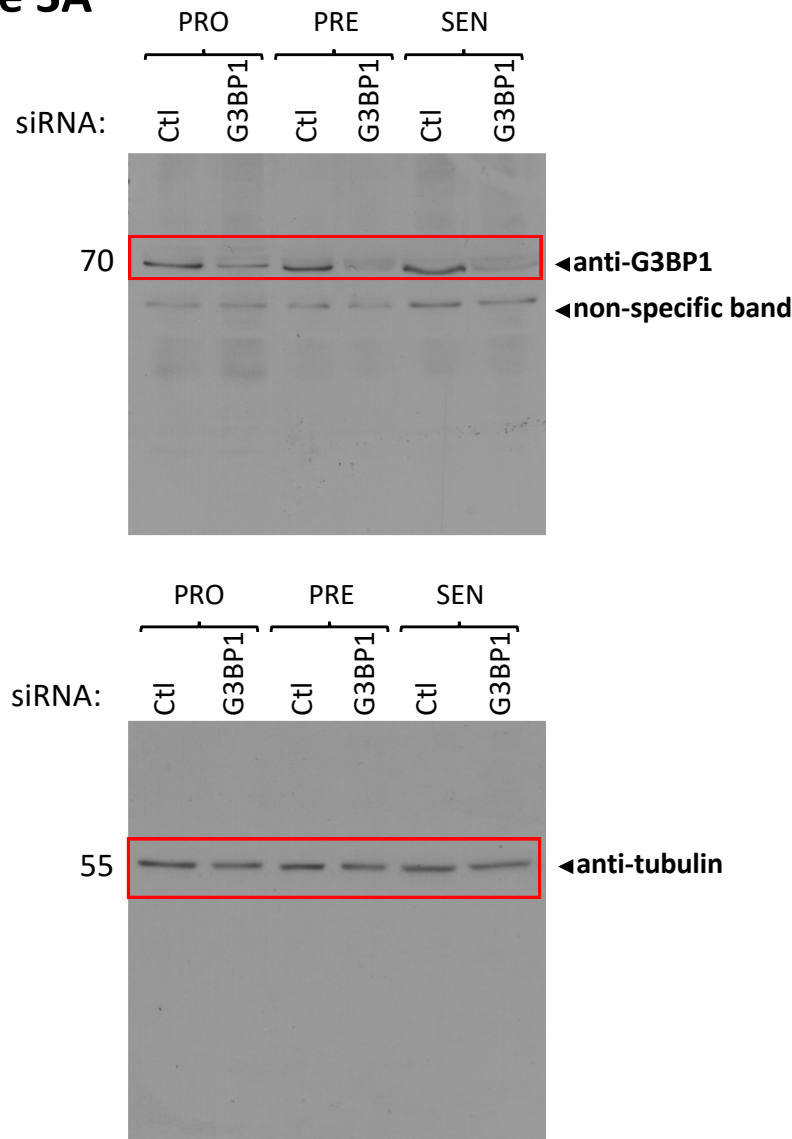

Supplement: Supplementary file 8 — Source Data for Figure 3 [file EMBR-19-e44722-s007.pdf]

Figure 6

Figure 6A

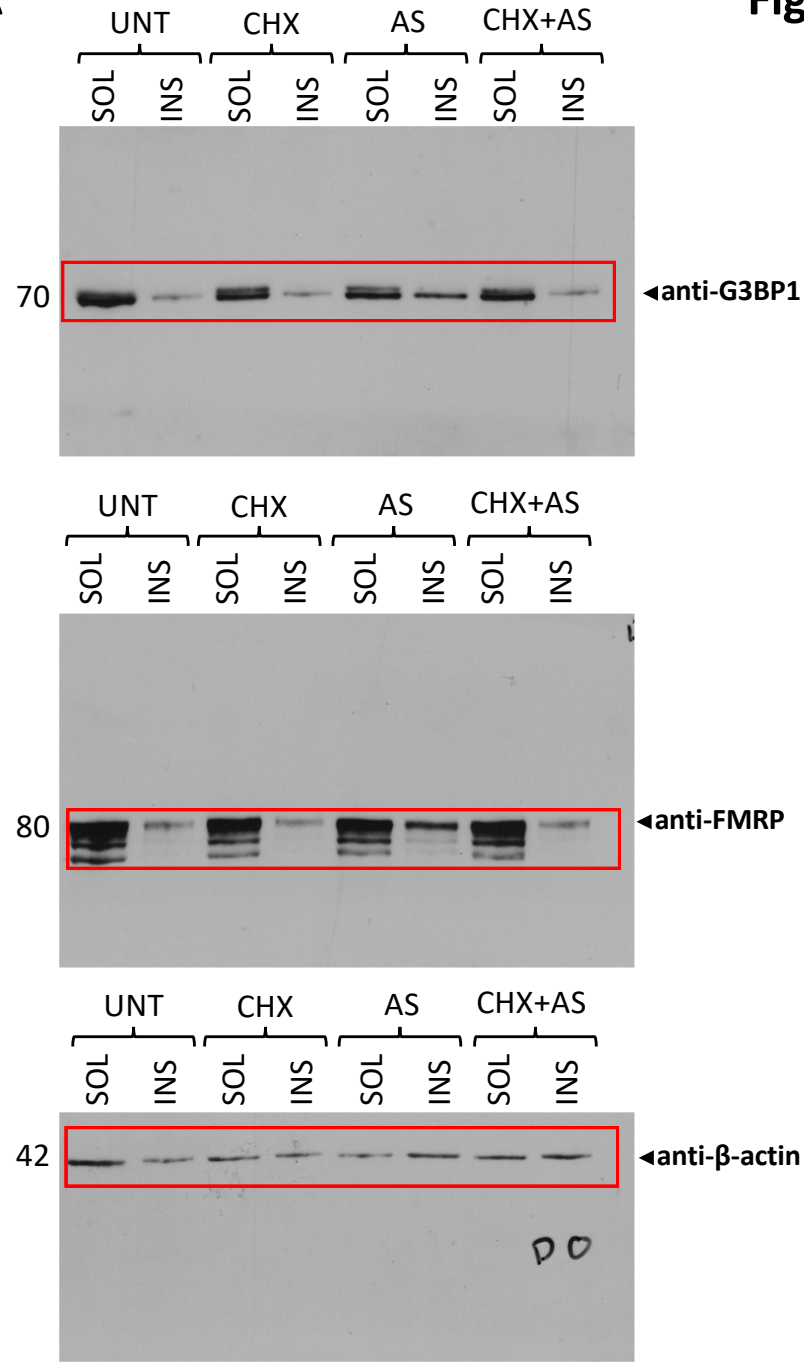

Figure 6D

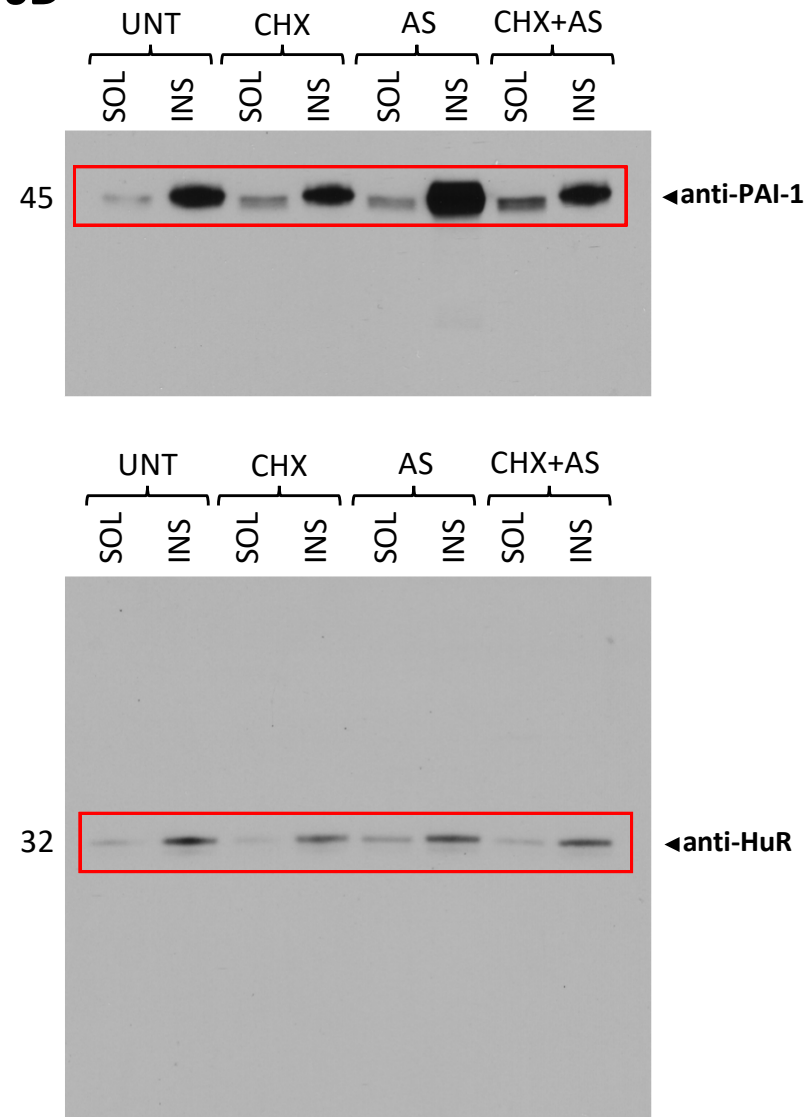

Supplement: Supplementary file 9 — Source Data for Figure 6 [file EMBR-19-e44722-s008.pdf]

Figure 7

Figure 7A

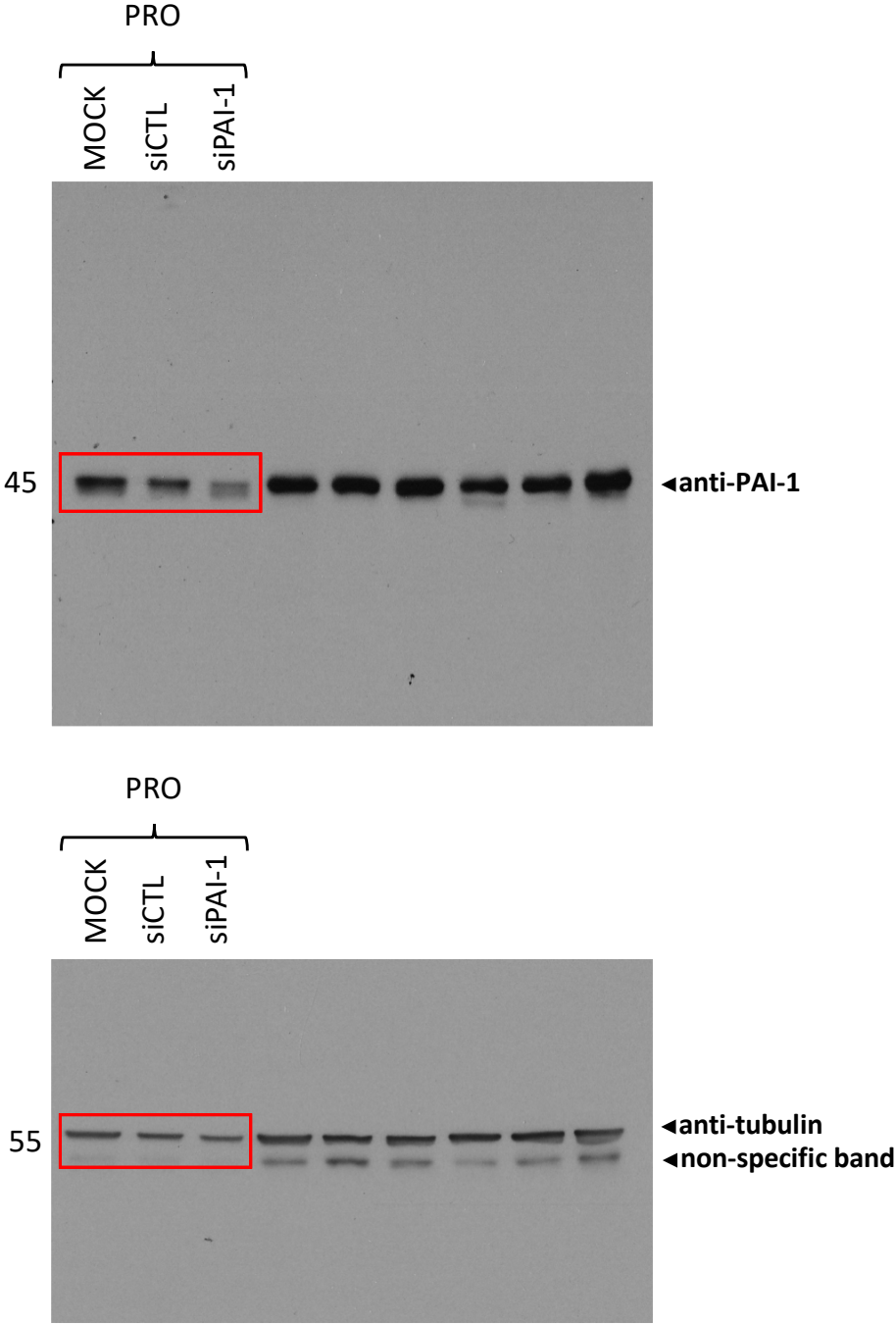

Supplement: Supplementary file 10 — Source Data for Figure 7 [file EMBR-19-e44722-s009.pdf]

Figure 8

Figure 8D

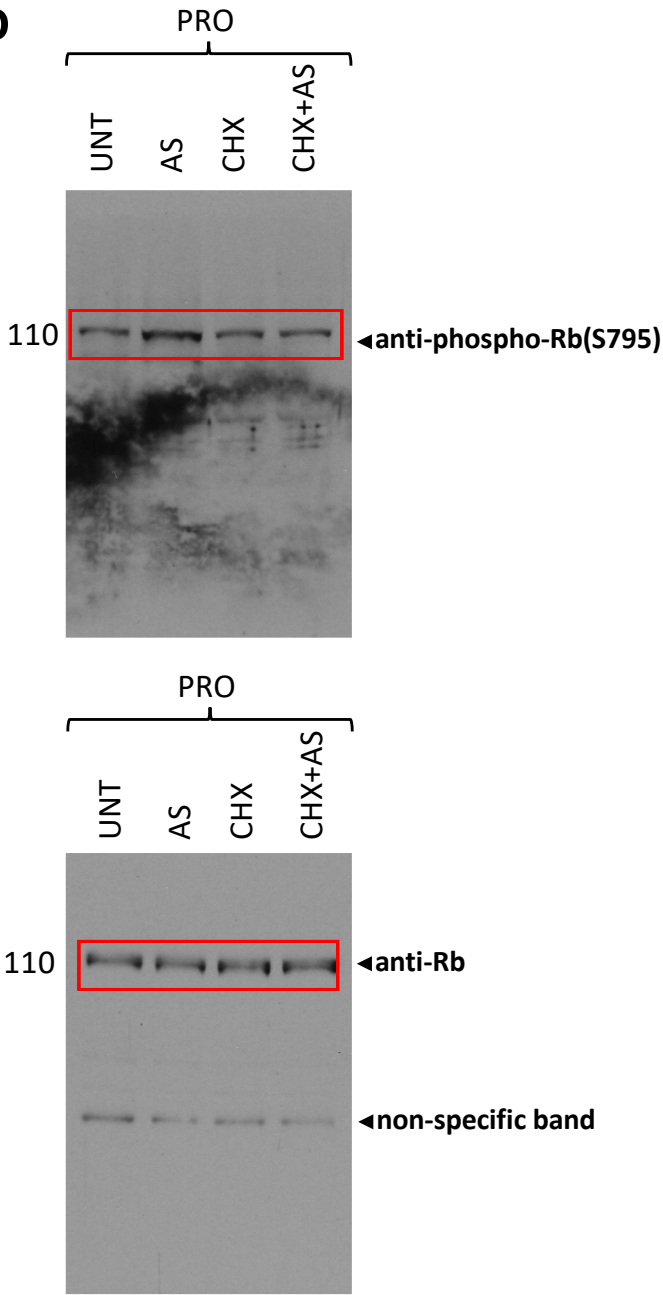

Supplement: Supplementary file 11 — Source Data for Figure 8 [file EMBR-19-e44722-s010.pdf]
